# Cross-feeding creates tipping points in microbiome diversity

**DOI:** 10.1073/pnas.2425603122

**Published:** 2025-05-06

**Authors:** Tom Clegg, Thilo Gross

**Affiliations:** ^a^Helmholtz Institute for Functional Marine Biodiversity at the University of Oldenburg, Oldenburg 26129, Germany; ^b^Alfred Wegner institute, Bremerhaven 27570, Germany; ^c^Institute for Chemistry and Biology of the Marine Environment, University of Oldenburg, Oldenburg 26111, Germany

**Keywords:** complex networks, microbial ecology, percolation theory, tipping points

## Abstract

A key unresolved question in microbial ecology is how the extraordinary diversity of microbiomes emerges from the interactions among their many functionally distinct populations. This process is driven in part by the cross-feeding networks that help to structure these systems, in which consumers use resources to fuel their metabolism, creating by-products which can be used by others in the community. Understanding the effects of cross-feeding presents a major challenge, as it creates complex interdependencies between populations which can be hard to untangle. We address this problem using the tools of network science to develop a structural microbial community model. Using methods from percolation theory, we identify feasible community states for cross-feeding network structures in which the needs of consumers are met by metabolite production across the community. We identify tipping points at which small changes in structure can cause the catastrophic collapse of cross-feeding networks and abrupt declines in microbial community diversity. Our results are an example of a well-defined tipping point in a complex ecological system and provide insight into the fundamental processes shaping microbiomes and their robustness. We further demonstrate this by considering how network attacks affect community diversity and apply our results to show how the apparent difficulty in culturing the microbial diversity emerges as an inherent property of their cross-feeding networks.

## Significance

Understanding how diversity is maintained in microbial communities presents a significant challenge, as cross-feeding networks create complex interdependencies between consumer populations that can be hard to disentangle. We address this problem by using methods from network percolation theory to develop a model that captures the dependence of microbial community diversity on cross-feeding network structure. Our results identify tipping points at which small structural changes can trigger the collapse of cross-feeding networks, leading to catastrophic loss of diversity. Furthermore, we demonstrate how perturbations to cross-feeding networks affect diversity, showing how the difficulty of culturing diverse microbiomes may arise from the structural constraints of their interaction networks. These findings offer insights into the fundamental mechanisms shaping microbiomes and their robustness.

Microbiomes are among the most diverse ecological systems on Earth, consisting of hundreds of functionally distinct populations interacting in complex networks of resource consumption and exchange (1–3). They are ubiquitous and perform many vital functions, from the cycling of nutrients in ecosystems (4) to the mediation of gut health (5). As advances in sequencing technologies allow us to examine microbiomes in increasing detail, there is a growing interest in answering fundamental questions about the ecology of the systems and how they maintain their extraordinary functional and taxonomic diversity (especially relative to macroscopic ecological communities) (6–9). Of particular importance are their cross-feeding dynamics, in which microbial consumers secrete the by-products of their metabolism into the environment, allowing them to be used by other members of the community (10). Cross-feeding has been shown to be widespread and helps to maintain both the high diversity and varied functional capacity of microbiomes (11, 12).

Although microbial communities are important, untangling their inherent complexity is difficult. In response, a recent surge of work has successfully applied methods from theoretical ecology to address this problem (13–18). This work is based on the insight that community-level properties are emergent, arising from the pattern of interactions between populations rather than from any individual component (19). By linking the structure of interaction networks to system-level properties, this approach has been successfully applied to study the functioning and stability of complex ecological systems (20–23). In microbiomes, previous studies have used this rationale to explore different aspects of cross-feeding, including its effect on robustness to disturbance (24) and its role in driving different regimes of community organization (16).

Viewing microbiomes through the lens of ecological theory also raises new questions. The stability of complex communities has been a central focus in ecology for over a century (25–28). One key aspect is the presence of critical transitions, where changes to structural parameters result in shifts in system-level properties such as community diversity (29). These transitions can be continuous, though particular interest has been paid to those in which long term behaviors change abruptly, referred to here as tipping points. Although tipping points are well documented in simpler systems, such as the clear-turbid transition in lakes (30), their role in complex and diverse communities like microbiomes is less clear (31, 32).

One phenomenon that tipping points may help explain is the widespread unculturability of microbial diversity. Attempts to culture microbes typically capture only a small proportion of natural diversity, prompting the question: Why do communities collapse to low-diversity states in the lab? (33) Lab cultures tend to provide abundant energy sources, reducing the pressure of resource competition. Thus, competition alone is unlikely to cause this collapse. Instead, it may result from the absence of specific metabolites produced by populations not captured in the sample. This represents a structural constraint, as networks of metabolic dependencies limit population persistence and overall community diversity. The breakdown of cross-feeding interactions has been suggested as a key factor in microbial culturability (33), supported by evidence of widespread auxotrophies in microbes (the inability to synthesize essential metabolites), as well as the demonstrated success of coculture techniques in growing otherwise unculturable strains (12, 34).

If microbial diversity is shaped by structural interdependencies, then understanding how cross-feeding network breakdown leads to diversity loss requires an approach that can capture these cascading effects. This type of problem has long been studied in the context of network percolation, a framework that describes how structural dependencies within networks influence system-wide connectivity and function. Percolation-based approaches have been widely used in network science (35–38), providing a suite of tools, such as the generating function formalism (39). In the case of microbiomes, metabolic interdependencies can be represented as a network of microbes and metabolites linked by edges representing the processes of consumption and secretion. By applying network percolation methods, we can quantify how the structure of cross-feeding interactions mediates community diversity and drives shifts between community states.

In this paper, we apply methods from network science to understand how cross-feeding networks mediate the maintenance of diversity in complex microbiomes. We formulate a simple, generic model of a microbial community, inspired by recent advances in consumer–resource theory (15, 16), that captures cross-feeding interactions between consumer populations. The exchange of metabolites leads naturally to a network representation of the community structure, which we analyze utilizing tools from network percolation theory. We show how consumer persistence within the community can be defined in terms of simple statistical features of their cross-feeding network, letting us link interaction structure to emergent diversity. Furthermore, we show that the model has tipping points at which the community abruptly transitions between states of high and low diversity. Finally, we show how tipping points provide insight into the challenge of culturing microbial diversity, illustrating how the structure of interaction networks governs community robustness and responses to perturbation.

## Results

### The Microbial Community Hypergraph model.

**Fig. 1.**
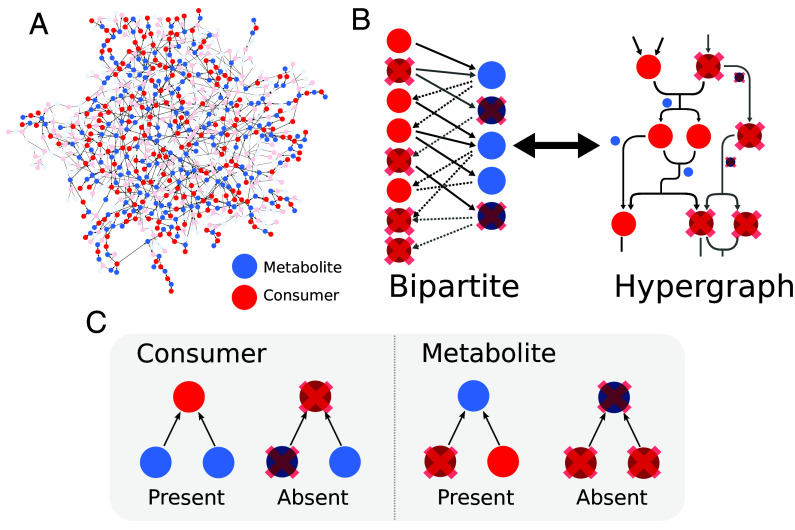
Cross-feeding networks and the microbial community model. Diagram showing the overall structure of the microbial community model. (*A*) A microbial community with a complex random cross-feeding network. Nodes represent consumers (red) and metabolites (blue) with links indicating consumer requirements M→C and metabolite production C→M. (*B*) The network has alternative representations as a bipartite graph or a hypergraph in which consumer nodes are linked by directed hyperedges representing metabolite uptake and release. Consumers and metabolites are marked present or absent (nodes with a cross) based on the following rules. (*C*) Simple rules dictate the presence of populations and metabolites. Consumers are present when all their required metabolites are present (*Left*). Metabolites are present when any one of the producers is present (*Right*).

We consider a complex community of *N* microbial consumer populations interacting through the consumption and exchange of a set of *M* metabolites (Fig. 1). Following ref. 15, we assume that each population requires a fixed set of metabolites to persist in the system. As a result of their metabolic activity, each population also produces a set of metabolites, which are released by continuous secretion or upon the death of the cell. We define each population entirely in terms of its functional role which is determined by the metabolites it requires and produces. The model can be imagined as a directed bipartite network of populations and metabolites with two sets of links representing metabolite consumption and production. Alternatively, we can represent the network as a hypergraph in which nodes are consumer populations linked by directed hyperedges representing metabolite production and consumption (Fig. 1*B*). For clarity, we will use the bipartite representation throughout the rest of the paper.

For simplicity, we do not model the dynamics of the system and focus entirely on the structural feasibility of cross-feeding networks. Each population and metabolite is described by a binary presence/absence variable (Fig. 1*C*). A metabolite is present if and only if at least one population that produces the metabolite is present. Conversely, a population is present if, and only if, all metabolites required by that population are present. With this approach, we do not aim to capture the full complexity of processes occurring in real microbial communities but rather provide an abstraction that allows us to build intuition about the effects of cross-feeding network structure. By focusing on cross-feeding interactions (as opposed to competition) we are able to derive results on how the diversity that communities can support is affected by the structural constraints present within cross-feeding networks.

We consider a statistical ensemble of such models in which the number of metabolites required by populations and the number of producers of each metabolite are drawn from the probability distributions *c*_*k*_ and *m*_*k*_ such that, for example, *m*_3_ is the probability that a metabolite is produced by three populations. By selecting *c*_*k*_ and *m*_*k*_ appropriately, we can emulate the desired features of a microbial community such as the relative numbers of currency metabolites (i.e. those required by many populations) or common by-products. We study the full ensemble of networks consistent with these distributions.

Below, we show that the model exhibits structural tipping points, reminiscent of the appearance of the giant component in simple graphs (40). We analyze this behavior by building on an elegant formalism (39), which uses mathematical objects called generating functions to store and manipulate sequences of numbers (*SI Appendix*, *Generating Functions*). These generating functions capture the structure of the network by encoding the distributions of the number of consumer requirements and metabolite producers. For the present model, the generating functions are defined as

[1] $$\begin{array}{c} C\left(x\right)=\sum c_{k}x^{k}\;\;\;M\left(x\right)=\sum m_{k}x^{k}, \end{array}$$

where C(x) encodes the number of requirements across consumers, M(x) represents the number of producers of each metabolite and, *x* is an abstract variable introduced to allow manipulation of the degree distribution (*SI Appendix*, *Generating Functions*).

Using these generating functions, we can determine the proportions of populations $c^{*}$ and metabolites $m^{*}$ that persist in the community. These quantities can be interpreted as the chance a randomly selected node is able to persist based on its requirements or producers. In ecological terms these represent the relative diversity of the system, the proportion of consumers able to persist in the cross-feeding network of all possible consumers in the community. We start by considering the conditions for persistence: Consumers must have all their requirements met, while metabolites need only one producer. We can express these probabilistically using the generating functions in Eq. **1** (*Materials and Methods*; *SI Appendix*, *Community Diversity*), leading to

[2] $$\begin{array}{c} \begin{array}{cc} c^{*} & =C(m^{*}),\\ m^{*} & =1-M(1-c^{*}). \end{array} \end{array}$$

From Eq. **2**, we can solve for $c^{*}$ and $m^{*}$, which define the stable configurations of communities in which the needs of consumers are met through the cross-feeding network. Though it appears simple, this expression captures the complexity of the interdependencies between populations and how consumers rely on the populations they interact with, who, in turn depend on their own interacting partners and so on. Crucially, it does so in terms of the statistical properties of the network, allowing us to link the structure of interactions between populations to the emergent diversity that the community cross-feeding network can support.

### Structural Tipping Points and Diversity.

To explore the patterns of diversity and community structure, we consider random cross-feeding networks in which metabolite requirements and production are distributed across the community. These random networks are reminiscent of Erdős–Rényi networks (41), and produce some interesting results due to the structure imposed by the microbial community model. Currently, there is no established consensus on the actual distribution of metabolite requirement and production in microbial communities. In the absence of this information, random networks provide a suitable and analytically tractable null model, allowing us to build a general intuition about the effects of network structure in large complex systems like microbial communities (42). We also note that random networks are far from a degenerate case and the intuition developed should be broadly applicable to other network structures.

In random networks the numbers of metabolite requirements and producers follow a Poisson distribution with generating functions $C\left(x\right)=\exp\left(z_{c}\left(x-1\right)\right),M\left(x\right)=\exp\left(z_{m}\left(x-1\right)\right)$, where *z*_*c*_ and *z*_*m*_ are the average number of consumer requirements and metabolite producers respectively. Applying these to Eq. **2** yields the self-consistency equation

[3] $$\begin{array}{c} c^{*}=\exp\left(-z_{c}\exp\left(-z_{m}c^{*}\right)\right). \end{array}$$

**Fig. 2.**
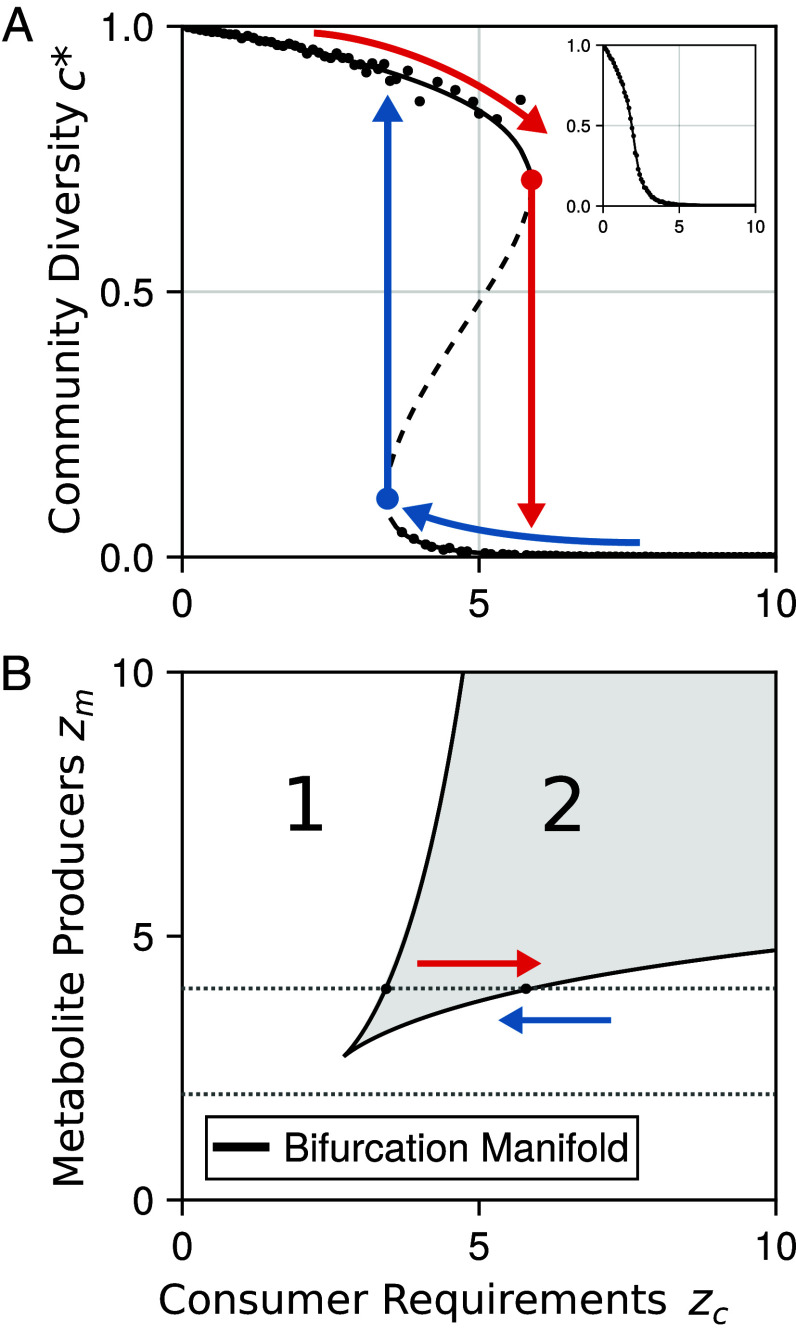
Cross-feeding networks have tipping points in consumer diversity. (*A*) Bifurcation plot showing the change in diversity as consumer requirements *z*_*c*_ varies and $z_{m}=4$. Lines show the predicted values from Eq. **3**, with solid lines indicating stable states and dashed lines unstable. Each point is from a randomly generated network with given structural properties with N=M=10,000. Arrows indicate the path dependence in the system. Increasing *z*_*c*_ (red arrows) leads us over the tipping point from a high to low diversity state while decreasing (blue arrows) does the opposite. The *Inset* plot shows the same for a value of $z_{m}=2$, where there is no tipping point and the change in diversity is continuous. (*B*) Phase plot showing the arrangement of tipping points and diversity in the two-dimensional parameter space. Changes in diversity are continuous in the white area (1). The points at which transitions happen form the bifurcation manifolds (black lines). The shaded area (2) indicates the region of bistability and path dependence where high and low diversity states are possible depending on the history of the system. The dotted lines indicate the slices over which (*A*). is displayed and the arrows the path dependence.

Solving Eq. **3** for $c^{*}$ reveals how the stable level of consumer diversity varies depending on the distribution of consumer requirements and metabolite producers characterized by *z*_*c*_ and *z*_*m*_ (Fig. 2 and *SI Appendix*, Fig. S1). Overall, community diversity increases as a function of the average number of metabolite producers *z*_*m*_, but falls with increasing consumer requirements *z*_*c*_. This relationship makes sense, since more consumer populations are able to persist when their requirements are more likely to be produced or when they have fewer requirements in the first place.

The change in diversity with network structure is initially continuous, shifting between high and low diversity states as described above (Fig. 2*A*, *Inset* plot). However, if either consumer requirements *z*_*c*_ or the number of metabolite producers *z*_*m*_ is varied sufficiently, discontinuous transitions become possible and tipping points appear. For example, if we fix *z*_*m*_ to 4 and slowly increase consumer requirements *z*_*c*_ from 0, community diversity gradually falls until a tipping point is encountered (known as fold bifurcations in dynamic systems theory) and the system enters a low diversity state (Fig. 2*A*). This behavior is driven by the collapse of the cross-feeding network. Increased requirements result in the loss of consumers which causes the loss of the populations dependent on their metabolites, creating a cascading effect. Conversely, if we take a community and reduce *z*_*c*_ from above, it will remain stuck in the low diversity regime, even as it passes the original tipping point, until a second tipping point is reached, and the community jumps back to the high diversity state. In this case, reductions in requirements allow the persistence of populations who produce metabolites others need, allowing the cross-feeding network to reassemble. Between these two transition points lies a region of path dependence or hysteresis, where the system can be either in a state of high or low diversity, depending on its history.

Considering variation in both consumer requirements *z*_*c*_ and metabolite producers *z*_*m*_ simultaneously results in a two-dimensional phase space in which transitions can occur (Fig. 2*B*). In this space the tipping points form two curves marking the points at which the high- and low-diversity states appear, also known as bifurcation manifolds. These curves eventually meet at a single point, $z_{m}=z_{c}=e$ and annihilate in a cusp bifurcation, which acts as an organizing center for the tipping point behavior.

Both the bifurcation manifolds and the cusp point can be derived analytically and agree with numerical solutions to Eq. **3** (*SI Appendix*, *Tipping Points and Bifurcation Analysis*). These results are robust to additional structural features, such as correlations in network structure (*SI Appendix*, *Correlated Degrees* and Figs. S2 and S3). We also validated these predictions with simulated microbial cross-feeding networks, which showed near perfect agreement with the theoretical predictions, including the expected patterns in diversity and tipping point behavior (*Numerical Simulations*).

### Microbial Community Robustness and Culture.

To illustrate the tipping point and further explore the robustness of community diversity to changes in network structure, we next apply our model to the question: Why are we unable to culture so much of the microbial diversity we see in nature?

**Fig. 3.**
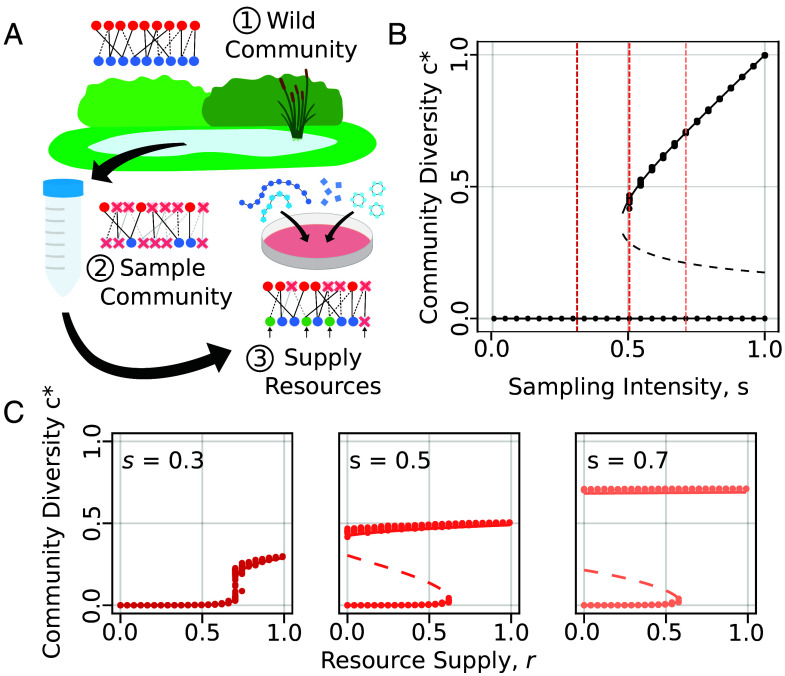
Cultured community diversity collapses due to tipping points in cross-feeding networks. (*A*) Diagram showing how sampling a wild community and culturing on a given medium affects cross-feeding network structure in the model. From a wild community (1), sampling removes some proportion of consumer populations and any metabolite they produce (2). Supplying resources in culture allows the partial recovery of the cross-feeding network (3), resulting in the final community. (*B*) Plot showing how community diversity $c^{*}$ increases as a function of the intensity of sampling *s*. Lines show analytical solutions from Eq. **3**, solid lines are stable and dashed unstable. Points show solutions obtained from randomly generated networks with [mean degree $z_{c}=z_{m}=10.0$,] N=M=1,000 and 100 replicates at each value of *s* (or *r* below). Dashed lines indicate the values of *s* in the plots below. (*C*) Plots showing the final community diversity $c^{*}$ against resource supply *r* across three levels of *s*. Again lines and points show solutions from analytical and numerical simulations respectively.

We approach this problem by considering how the culture of microbial communities acts as a perturbation to their cross-feeding networks and the diversity they are able to support (Fig. 3*A*). We represent this process using our model in two steps. First, we sample a community from the environment, collecting only a proportion of the populations present. Second, we culture the community in medium, supplying it with some of the resources necessary for growth (henceforth referred to as externally supplied resources). This procedure not only acts as a direct perturbation to communities, removing consumers and supplying resources, but also has indirect effects, as the loss and gain of consumers and metabolites propagates the cross-feeding network.

While enumerating the effects of these perturbations on individual populations is challenging, the use of generating functions simplifies the problem. In our model, we can encode the sampling and the external supply of resources in their own functions S(x)=1+s(x−1) and R(x)=x+r(1−x), where *s* and *r* are the proportion of consumers captured in the sample and the proportion of externally supplied resources respectively. Combined with Eq. **1** these functions allow us to express the structure of the cross-feeding network (subscript A) after sampling and culture succinctly in a new set of generating functions (*SI Appendix*, *Community Robustness, Sampling and Culture*).

[4] $$\begin{array}{c} C_{A}\left(x\right)=C\left(R\left(x\right)\right)\;\;\;M_{A}\left(x\right)=M\left(S\left(x\right)\right). \end{array}$$

These generating functions capture the probabilities that a consumer or resource has a given number of requirements or producers in the final community after culture, accounting for the change in the average number of consumer requirements $y_{c}=\left(1-r\right)z_{c}$ and metabolite producers $y_{m}=\left(s\right)z_{m}$, due to the loss and gain of consumers and resources. These expressions can then be used to solve for the proportion of populations and metabolites present, similar to Eq. **2**.

Fig. 3 *B* and *C* show how the process of sampling and culturing leads to the diversity of the final community. In the first stage, sampling a smaller proportion *s* of the wild community results in both reduced diversity and fewer metabolite producers *y*_*m*_. If our sampling is incomplete, too many producers may be lost, causing the community cross-feeding network to break down, shifting the community into the low diversity state. The subsequent external supply of resources in culture effectively reduces the number of metabolite requirements of consumers *y*_*c*_, which promotes the persistence of consumers and in turn, increases the relative diversity in the community. Depending on the structure of the system, the external supply of resources may allow communities to persist in the high diversity state. However, due to the path dependency discussed above, reassembling the community may be difficult and a greater proportion of resources must be externally supplied to counter the loss of metabolite producers via sampling.

Together these results illustrate one mechanism explaining why it is so difficult to culture much of the microbial diversity we see in nature: The inherent structure of cross-feeding networks can create tipping points that make them fragile to perturbation. If we sample insufficiently or if resource requirements are inadequately supplied, the community cross-feeding network is unlikely to remain active, leading to the loss of diversity. However, it is important to note that cross-feeding structure is not the only determinant of culturability. In reality, microbial persistence is influenced by multiple factors, including physical (i.e., non-metabolite-based) environmental conditions and microbial dormancy dynamics (34).

### Conclusion.

In this paper, we develop a model of microbial community structure, addressing the question of how the diversity of complex microbiomes emerges from the interactions of individual populations. By utilizing tools from network science, our results link the exchange and consumption of metabolites in cross-feeding networks to the emergent diversity and robustness of microbial communities. Importantly, our work reveals the existence of tipping points at which communities can jump between states of high and low diversity as cross-feeding networks collapse and reassemble.

The tipping points we observe represent an exciting advance in our understanding of microbial cross-feeding, identifying a potentially important mechanism that drives patterns of microbial community diversity. The existence of these behaviors in such a simple model suggests they may be generally applicable in systems structured in this way, where they likely interact with other factors such as competition or assembly dynamics (see below) to determine community diversity. The presence of tipping points and path-dependent dynamics also suggest an inherent fragility in the structure of microbiomes. We find that perturbations can cause the collapse of cross-feeding networks and their associated diversity and that recovery may be difficult due to the interdependencies they create between populations. These results provide a testable prediction: Perturbations, such as the removal of populations, should result in abrupt changes to diversity, as illustrated in our example of sampling and culture of communities. In principle, such experiments should be relatively easy to carry out and combined with our results, have the potential to improve our understanding of microbiomes and their robustness.

One of our major results is the identification of regions in network structure parameter space (*z*_*c*_ and *z*_*m*_) where tipping points occur. While our model ultimately represents an abstraction of microbial cross-feeding structure, we argue these tipping points are relevant for our understanding of real communities for two reasons. First, the regions of parameter space in which these behaviors occur are at a relatively low magnitude ($z_{c}=z_{m}=2.71...$), showing that consumers only need to use and produce a few resources to exhibit tipping point behavior. Given the ubiquitous nature of auxotrophies and obligate cross-feeding (10, 12), it is reasonable to assume that microbial communities operate somewhere within this area of parameter space. Second, our results on the culturability of microbial communities suggest that sampling effects can be understood as structural perturbations to cross-feeding networks, acting by scaling the effective numbers of producers and requirements. This implies that any community residing within the fold bifurcation region discussed above will always have a perturbation capable of shifting it across the tipping point, thereby altering community diversity.

Our work focuses on the structure of cross-feeding interactions and how they constrain consumer diversity, reflecting a conscious choice in our modeling approach. Although we do not account for competition explicitly, we argue that our model provides valuable insights by illustrating a mechanism likely at play in real communities, interacting with competitive effects to shape microbial diversity. We also note that the relative importance of these competitive and cooperative effects within microbial communities is likely not equal across different contexts (43) and that there are some scenarios where the structural constraints of cross-feeding networks (and associated tipping points) will be more relevant. For example, one observation from empirical studies is the tendency of communities to become less competitive over time as auxotrophies develop and communities become more dependent on obligate cross-feeding interactions (44–46). In this case, we might expect our results to be more relevant in communities in more stable environments where these cross-feeding networks have time to develop and act as more of a constraint on community structure.

Our structural approach does not account for other dynamic processes known to influence microbial community diversity, such as assembly dynamics (i.e. immigration) and metabolic plasticity, both of which alter network structure over time. In principle, these processes could be incorporated into our model by using the generating function formalism, an approach previously applied to study network dynamics in other contexts, such as epidemic spreading (47). In a microbial context, this would allow us to explore how consumers dynamically adapt their metabolism to changing environments or competition via immigration-extinction dynamics, offering promising directions for future research.

Overall, our results provide an example of a tipping point in a highly diverse and complex ecological system. Most prior examples of ecological tipping points focus on simple systems with relatively few interacting components, not on diverse and complex systems like those considered here. There is ongoing debate about whether such systems undergo tipping points and how useful the concept is in understanding biodiversity decline (31, 32). Our results contribute to this discussion by demonstrating a well-defined tipping point and showing how it can be triggered through community disturbances. Identifying how the structural mechanisms we describe relate to ecological robustness more broadly is an exciting direction for future research, with the potential to enhance our understanding of biodiversity loss.

## Materials and Methods

### Community Diversity.

In order to determine the proportion of populations $c^{*}$ and metabolites $m^{*}$ able to persist in the community, we first determine the probability that a randomly selected consumer population is present. This is the same as asking what is the chance we select a consumer with *k* requirements and that those *k* metabolites are also present leading to

[5] $$\begin{array}{cc} c^{*} & =\sum c_{k}{\left(m^{*}\right)}^{k}. \end{array}$$

We can also apply the same approach to the metabolites, asking what is the chance we select a metabolite with *k* producers and that at least one of these is present

[6] $$\begin{array}{cc} m^{*} & =\sum m_{k}\left(1-{\left(1-c^{*}\right)}^{k}\right)\\ & =1-\sum m_{k}{\left(1-c^{*}\right)}^{k}, \end{array}$$

where we have used the fact that the probabilities sum to 1, $\sum m_{k}=1$ in the last step.

Eqs. **5** + **6** both have forms identical to the generating functions defined in Eq. **1**. This lets us write the system in terms of the generating functions leading to

[7] $$\begin{array}{c} \begin{array}{cc} c^{*} & =C(m^{*}),\\ m^{*} & =1-M(1-c^{*}). \end{array} \end{array}$$

which is equivalent to Eq. **2** in the main text. From this set of equations it is easy to exclude $m^{*}$ and obtain a self-consistency equation

[8] $$\begin{array}{c} c^{*}=C\left(1-M\left(1-c^{*}\right)\right), \end{array}$$

which can be solved for $c^{*}$ given a specific set of network degree distributions encoded in the generating functions C(x) and M(x). The solutions to Eq. **8** give the proportion of total consumer populations that can persist and thus the relative diversity in the community. With the value for $c^{*}$ we can also substitute back into Eq. **7** and obtain the corresponding values of $m^{*}$.

### Numerical Simulations.

In order to verify the analytical predictions of Eq. **3**, we generated random microbial cross-feeding networks and determined the proportion of persisting consumer diversity based on the rules discussed in the main text (48).

To generate random network structures with defined distributions of consumer requirements and metabolite producers, we use a configuration model type approach (49). In these networks, we consider directed links to match the flow of resources, i.e. consumption is represented by links from metabolites to consumers and metabolite secretion from consumers to metabolites.

We start by initializing the system with *N* consumer populations and *M* metabolites. We then draw their respective numbers of requirements and producers from Poisson distributions with the average values *z*_*c*_ and *z*_*m*_, thus defining the end points of each link in the network. In order to construct the whole network we next need to place the start point of each link. As the total number of start and end points must be equal, this places a additional constraint on the average in- and outdegree such that $z_{m}^{\mathrm{in}}=az_{c}^{\mathrm{out}}$ and $z_{c}^{\mathrm{in}}=az_{m}^{\mathrm{out}}$, where a=M/N (*SI Appendix*: eq. 34). To sample the number of links starting at each node, we therefore need to sample from a Poisson distribution with the correct mean, conditioned on the total number of links in the network. This follows a multinomial distribution with *S* trials and *n* events, where *S* is the number of links and *n* is the number of nodes we want to partition them over. Once we have the number of start and end points for each node, we randomly wire the network by aligning the start and end points of each link and then shuffling them to distribute the links across the network.

Once we have a network structure, we determine the proportion of consumers persisting with a simple iterative approach. We initialize the system by marking a random proportion of consumers and metabolites present. We then move through all nodes in the network in random order, updating their presence based on the rules discussed in the main text. After every loop, we check to see whether the state of any node in the network has changed. Over multiple iterations the system converges to a steady state, in which the only consumers present are those whose needs are met by the metabolites available and vice versa. We stop the process once the node states converge and no longer change. The proportion of persisting consumers and metabolites can be calculated as the ratio of the number present in the final state relative to the total number in the network.

## Supplementary Material

Appendix 01 (PDF)

## Data Availability

Code to generate results and figures is available in the Zenodo repository (48).
