# Supplementary material for: Cross-feeding creates tipping points in microbiome diversity
Source: Proc Natl Acad Sci U S A. 2025 May 6;122(19):e2425603122. doi: 10.1073/pnas.2425603122 (PMC12088387; doi:10.1073/pnas.2425603122)
Supplement: Supplementary file 1 — Appendix 01 (PDF) [file pnas.2425603122.sapp.pdf]

Supporting Information for

Cross-feeding Creates Tipping Points in Microbiome Diversity

Tom Clegg, Thilo Gross

Tom Clegg, thomas.clegg@hifmb.de

This PDF file includes:

Supporting text
Figs. S1 to S3
SI References

Supporting Information Text

Generating Functions. Generating functions (GF) are mathematical objects that allow us to represent sequences of numbers as coefficients in a power series. GFs are invaluable when working with complex networks as they allow us to deal with the combinatorial explosions that often follow when we try to enumerate though the many possible connections in a network. In this section we will provide a basic overview of their properties and application (for a more complete review for their application to networks see (1)).

As seen in the main text a generating function for the distribution of a discrete random variable a is defined as

$$G(x) = \sum p_k x^k \qquad [1]$$

where x is an arbitrary complex variable and p_k is the probability mass at the value k which must sum to one over the support of $a \in \mathcal{A}$ such that $\sum_{k\in\mathcal{A}} p_a(k) = 1$. In terms of the generating function this is expressed as

$$G(1) = \sum p_k = 1 \qquad [2]$$

which is called its norm. We can also recover the point probabilities from a generating function by taking the correct terms of the series expansion

$$p_k = \frac{G^{(k)}(0)}{k!}. \qquad [3]$$

Calculating the moments of the distribution is simple too, for example to calculate the mean we simply take the first derivative and evaluate at 1

$$G'(1) = \sum_k p_k k = \langle k \rangle . \qquad [4]$$

This can also be generalised to higher moments

$$\langle k^n \rangle = \sum_k p_a(k) k^n = (kG'(1))^{(n)} \qquad [5]$$

where we iteratively take the derivative and multiply by k n times.

Some of the most powerful features of generating functions are the operations we can preform on them. For example they can be used to easily calculate the sum of two discrete random variables. To illustrate this it is useful to consider an example where we we have two identical four sided dice and we want to compute the distribution of their sums. The generating function for each die is identical and can be written as

$$G_{\text{die}}(x) = p_1 x + p_2 x^2 + p_3 x^3 + p_4 x^4 \qquad [6]$$

We can first attempt the calculation manually, enumerating the ways we can arrive at each total sum. For example to arrive at the value of 4 we can either roll two 2s or a 1 then a 3 or a 3 then a 1 giving a total probability of $p_2 p_2 + 2p_1 p_3$. Writing out the full equation quickly realise that the coefficients follow the combinatorics of polynomials and that we can write the generating function for the sum of the two die as

$$G_{2\text{die}} = \left(p_1 x + p_2 x^2 + p_3 x^3 + p_4 x^4\right)^2 = G_{\text{die}}(x)^2. \qquad [7]$$

Thus, the generating function of the sum of any two random variables can be calculated simply multiplying their respective generating functions, avoiding the need for cumbersome enumeration through all possible values it can take.

Generating functions also allow the enumeration of sequential events in the "dice of dice" rule which is useful when considering network structure. For example imagine a scenario in which we want to roll our 4-sided dice to determine how many times we flip a coin. What is the distribution for the total number of heads? To address this we can first write a generating function for our coin denoting tails as 0s and heads as 1s

$$G_{\text{coin}}(x) = c_0 + c_1 x. \qquad [8]$$

Again, we can try to write the generating function for the total manually, enumerating through all possible combinations. First we consider the case where you get a one on the die roll with probability p_1, this means we get one coin toss, which is generated by G_{coin}. With a probability p_2 we roll a two and get two coin tosses, but applying the rule above

we know that the sum of two coin tosses is generated by $(G_{\text{coin}})^2$. The same rule can be used for rolls of 3 and 4, and putting this all together gives us the generating function

$$\begin{aligned} G_{\text{game}}(x) &= p_1 G_{\text{coin}}(x) + p_2 {G_{\text{coin}}}^2(x) + p_3 {G_{\text{coin}}}^3(x) + p_4 {G_{\text{coin}}}^4(x) \\ &= G_{\text{die}}(G_{\text{coin}}(x)) \end{aligned} \qquad [9]$$

The second line shows that the result of this game can be generated by applying the generating function of the die to the generating function of the coin toss. This is the dice-of-dice rule: If we are summing over a random number random events then the generating function for the outcomes is the generating function of the events plugged into the generating function for the number of these events.

The Microbial Model. In this section we show the derivation of the microbial model in more detail, discussing the discontinuous percolation transition and extensions of the model to correlated consumer and metabolite degree.

As discussed in the main text we consider a community of N microbial populations and M metabolites. Each consumer has a set of resources that they require to persist, the number and identity of which varies amongst species. A consumer population is able to persist if all the resources it requires are present. It will use these resources to fuel growth and reproduction creating metabolic by-products in the process. Metabolites are present in the system if any of the consumer populations that produce them are present.

The model above has multiple interpretations as a network. We can consider the system a bipartite graph where the two components, populations and metabolites, are connected by two sets of directed links representing the requirements and production of resources respectively. Alternatively we can consider a directed hypergraph representation where the consumer nodes are linked via their connections to specific metabolites. In this framework each metabolite represents a directed hyperedge linking the producers of the metabolite to its consumers. Following the rules describe above, nodes in the hypergraph will be active only if all incoming hyperedges are present but edges can be activated by any source node.

The microbial communities model can be investigated using the mathematics of generating functions discussed above. We first define two probability generating functions to describe the distribution of populations requirements and metabolite producers

$$C(x) = \sum c_k x^k \qquad M(x) = \sum m_k x^k \qquad [10]$$

where $C(x)$ and $M(x)$ are the generating functions for the numbers of consumer requirements and metabolite producers and c_k and m_k are the probabilities that a consumer or metabolite have indegree k respectively. Following the standard definitions of probability generating functions we can calculate important quantities such as the average number of requirements and metabolite producers by taking the derivative and evaluating at 1

$$z_c = C'(1) \qquad z_m = M'(1).$$

Community diversity We now ask what is the number of consumer populations and metabolites that can be supported in a community with a given distribution of metabolite requirements and producers (i.e. $C(x)$ and $M(x)$). Denoting the proportion of populations and metabolites present as c^* and m^* we first determine the probability that a randomly selected consumer population is present. This is the same as asking what is the chance we select a consumer with k requirements and that those k metabolites are also present leading to

$$c^* = \sum c_k (m^*)^k. \qquad [11]$$

We can also apply the same approach to the metabolites, asking what is the chance we select a metabolite with k producers and that at least one of these is present. This is equivalent to one minus the probability that all k producers of a metabolite are absent (given by $(1 - c^*)^k$) letting us write

$$\begin{aligned} m^* &= \sum m_k (1 - (1 - c^*)^k) \\ &= 1 - \sum m_k (1 - c^*)^k, \end{aligned} \qquad [12]$$

where we have used the fact that the probabilities sum to 1, $\sum m_k = 1$ in the last step.

We can see that Eqs. 11 theand 12 both have forms identical to the generating functions defined in Eq. 10. This lets us write the system in terms of the generating functions leading to

$$\begin{aligned} c^* &= C(m^*), \\ m^* &= 1 - M(1 - c^*). \end{aligned} \qquad [13]$$

From this set of equations it is easy to exclude m^* and obtain a self-consistency equation

$$c^* = C(1 - M(1 - c^*)), \quad [14]$$

which can be solved for c^*. The solutions to equation 14 give the proportion of total consumer populations that can persist and thus the relative diversity in the community. With the value for c^* we can also substitute back into Eq. 13 and obtain the corresponding values of m^*.

Random Networks. In this section we consider what proportion of the community is able to persist in a random network. In a random network populations require each metabolite with a fixed probability p_c and metabolites are produced by each consumer with a probability p_m. In the limit of a large network where $N, M \to \infty$ the numbers of consumer requirements and metabolite producers approach a Poisson distribution with parameters $z_c = Mp_c$ and $z_m = Np_m$, the average requirement and production degree. As they are Poisson distribution the generating functions $C(x)$ and $M(x)$ have the simple form

$$G(x) = \exp[z(x-1)] \quad [15]$$

where z is the average degree. Using Eq. 15 as the generating functions for population requirements $C(x)$ and metabolite production $M(x)$ yields

$$c^* = \exp\left[-z_c \exp\left(-z_m c^*\right)\right] \quad [16]$$

which can be solved to give the solutions for the proportion of consumer population present in the system c^*. These can in turn be used to solve for the metabolites m^*.

Tipping Points and Bifurcation analysis. We now consider how the solutions to Eq 16 give rise to tipping points in community diversity as the structure of cross-feeding network changes. Numerical solutions show that diversity within the community c^* undergoes a bifurcation once the average number of consumer requirements z_c and metabolite producers z_m pass as certain point (Fig.2, maintext). We can derive the points at which these transitions occur by determining the region of parameter space at which the solutions to Eq. 16 also have a zero-valued derivative.

We start by taking the derivative of Eq. 16 and setting it to zero

$$0 = z_c z_m \exp(-z_c \exp(-z_m c^*) - z_m c^*) - 1. \quad [17]$$

Values of z_c, z_m and c^* that satisfy Eqs. 16 and 17 form the bifurcation manifold where the tipping point behavior occurs. We next use Eq. 17 to get an expression for the c^* in terms of the two structural parameters by substituting Eq. 16 into Eq. 17

$$1 = z_c z_m c^* \exp(-z_m c^*)$$

which we then solve for c^*

$$-\frac{1}{z_c} = -z_m c^* \exp(-z_m c^*)$$

$$c^* = -\frac{W(-\frac{1}{z_c})}{z_m} \quad [18]$$

where $W(x)$ is the Lambert W function which gives the solution to equations of the form $y = xe^x$ as $x = W(y)$. For real values (such as the probabilities we consider here) the Lambert W function has two branches over the range $-1/e < x < 0$. Substituting this solution for c^* back into Eq. 16 gives an equation for the bifurcation manifold in terms of the structural parameters

$$z_m = -\frac{W(-1/z_c)}{\exp(W(-1/z_c)^{-1})}. \quad [19]$$

This expression gives the curve that marks the point of the tipping points in the model. It is made of up of two branches which mark the transitions between high and low diversity states in either direction. Fig. 2B (maintext) shows the arrangement of the manifolds, including the region in-between where multiple community states are possible depending on the history (i.e path dependency) in the system.

We can also use Eq. 19 to consider the point at which the bifurcation originates. As the Lambert W function is defined over the range when its argument $x > -1/e$. This means that the smallest value of z_c for which the bifurcation can occur is at $z_c = e$ which in turn gives the minimum value of $z_m = e$. Thus the cusp bifurcation, where the two manifolds marking the tipping points collide is at $z_c = z_m = e$

Community Robustness, Sampling and Culture. In this section we determine the effects of the sampling and culture of microbial communities through their cross-feeding network. We first consider how these processes are equivalent to the removal of nodes from the cross-feeding network and then derive the equations describing the state of the community after these attacks.

As discussed in the main text the process of sampling and culturing a microbial community can be broken into two stages in which we first incompletely sample the community, keeping only a proportion s of the populations. We then supply a proportion of the resources r that the microbes within may require. The final community consists of the populations that were initially sampled and then able to survive on the provided resources and any created as a result of secondary metabolism.

Considering the sampling of the community in the first step as an attack on the network is natural. When we sample we randomly miss a proportion $1 - s$ of the consumer populations who are then removed from the final community. The supply of resources has a similar, though more nuanced, interpretation. When a resource is supplied to the community it is present unconditionally. This means that any consumer needing this resource will automatically have this need fulfilled and we can remove all requirement links from the metabolite. The removal of all the links is equivalent to removing the node itself. Therefore the probability of supplying a proportion of the resources r is equivalent to removing them, and can be considered using the same techniques for attacks on the network.

Having established how attacks on the network are equivalent to the sampling and culture of communities we now consider how these processes affect network structure and ultimately the diversity that communities can support. It is useful to define an attack function for the node removals $A(x, a) = (1 - a) + ax$ which effectively represents a coin flip in which we keep a node with probability a and remove it with probability $(1 - a)$.

First we consider the consumer requirements in the final community. In the original community each consumer had needed k metabolites with probability c_k. After the attack we remove a proportion r of these needs by supplying them to the community. This is equivalent to flipping the attack coin k times letting us write the final generating function for the number of requirements as

$$C_{\mathrm{A}}(x) = \sum c_k(r - (1 - r)x)^k = C(A(x, 1 - r)). \quad [20]$$

The final expression can also be reached by following the dice of dice rule discussed in section x.

Second we consider the distribution of metabolite producers in the final community. This follows the same logic as above. In the original community a metabolite has k producers with probability m_k. Each of these k consumers has a probability s of being sampled in the final community. Again the total number of links in the final community is equivalent to flipping a coin k times and can be written as

$$M_{\mathrm{A}}(x) = \sum m_k((1 - s) - sx)^k = M(A(x, s)). \quad [21]$$

Note the attack depends on the probability s as we keep the proportion s in the final community and remove the rest. In the main text we define the two attack functions directly for simplicity of notation

$$S(x) = (1 - s) + sx = A(x, s) \quad [22]$$

$$R(x) = r + (1 - r)x = A(x, 1 - r) \quad [23]$$

With the generating functions for the structure of the network after the attack in hand it is now easy to derive the diversity in the final community after sampling and culture. The same derivation used to obtain Eq. 13 can be used on the generating functions after the attack $C_{\mathrm{A}}(x)$ and $M_{\mathrm{A}}(x)$ giving the new diversity in the community

$$c^* = C_{\mathrm{A}}(m^*), \quad [24]$$

$$m^* = (1 - M_{\mathrm{A}}(1 - m^*)), \quad [25]$$

After solving for c^* and m^* we apply corrections for the diversity lost and resources gained from the process directly

$$c^*_{\mathrm{A}} = sc^* \quad [26]$$

$$m^*_{\mathrm{A}} = r^* + (1 - r^*)m^*, \quad [27]$$

where consumer diversity after the attack, c^*_{A} is scaled by the s to account for the loss of species in the sampling stage and the proportion of metabolites is the sum of those supplied r plus any produced by consumers (second part scaled by $1 - r$).

Correlated Degrees. In this section we consider the extension of the model with correlations between the in- and outdegree of consumer populations and metabolites. It is important to note that these are *intranode* correlations, defining the relationship between the number of links entering and exiting consumer and metabolite nodes. For consumers this is between the number of resources they require and produce and for metabolites the number of populations they are produced by and required by. These correlations may arise if, for example, consumers that have many resource requirements also tend to produce a variety of metabolites (i.e. they have high metabolic diversity). Likewise metabolites that are produced by many populations may also tend to be used by many consumers representing the tendency of consumers to use resources available to them in the environment.

In order to include correlations we need to consider the joint in/outdegree distributions of nodes by using bivarate generating functions

$$C(x,y) = \sum_{jk} c_{jk} x^j y^k, \tag{28}$$

$$M(x,y) = \sum_{jk} m_{jk} x^j y^k. \tag{29}$$

These bivarate generating function follow the same basic principle as their univarate counterparts, representing the joint distribution of in- j and outdegrees k. The probabilities c_{jk} and m_{jk} encode the correlation between in- and outdegree for consumers and metabolites. The direction of edges follow the flow of material, metabolite consumption is represented by edges flowing out of metabolites and into consumers and vice versa.

We can derive a number of quantities from the generating functions including the marginal marginal distribution of in or out links

$$\begin{aligned} C^{\text{in}}(x) &= \sum_{jk} c_{jk} x^j 1^k = C(x,1), \\ C^{\text{out}}(x) &= \sum_{jk} c_{jk} 1^j y^k = C(1,y), \end{aligned} \tag{30}$$

as well as the marginal averages

$$z_c^{\text{in}} = \sum_{jk} c_{jk} j = \left. \frac{\delta C(x,y)}{\delta x} \right|_{x,y=1} \tag{31}$$

$$\tag{32}$$

It is also important to note that as we now explicitly track both in- and outdegree of each node we need to make sure the total degree across the network adds up. This places a constraint on the degree distributions such that the total number of start points for each type of link (representing a need or production relationship) equals the number of endpoints. This is separate from the intranode structure captured in the degree-correlations. These constraints on the degree distributions can be expressed in the equalities

$$\begin{aligned} \sum_i^N j_i &= \sum_a^M k_a \\ \sum_i^N k_i &= \sum_a^M j_a \end{aligned} \tag{33}$$

where j_i is the indegree of the ith consumer and k_a the outdegree of the ath metabolite. We can express also this in terms of the marginal averages by defining a constant α which gives the relative number of metabolites to populations $M = \alpha N$ and dividing through by N

$$\begin{aligned} z_c^{\text{in}} &= \alpha z_m^{\text{out}} \\ z_c^{\text{out}} &= \alpha z_m^{\text{in}}. \end{aligned} \tag{34}$$

Thus the average degrees are constrained by the relative size of the consumer and metabolite components. If they are equal $a = 1$ then the average degree must be the same.

In order to find the solutions for the proportions of consumers and metabolites present we follow a similar logic as before with one major difference. As the in- and outdegree are no longer independent we need to account for the

potential correlations in the node degrees. To make this adjustment we introduce an additional step, defining the probability of arriving a node following a random outgoing link backwards and it being present as c_1^* and m_1^* for consumer populations and metabolites respectively. We also need to define two new generating functions which give the indegree distribution of a node we arrive at by following a outgoing link backwards. This is similar to the concept of excess degree used in the giant component literature (2)

$$C_1(x) = \frac{\sum_{jk} c_{jk} x^j k}{\sum_{jk} c_{jk} k} = \frac{1}{z_c^{out}} \left. \frac{\delta C(x,y)}{\delta y} \right|_{y=1}. \qquad [35]$$

We also define the equivalent function for metabolites

$$M_1(x) = \frac{1}{z_m^{\text{out}}} \frac{\delta M(x,y)}{\delta y}. \Big|_{y=1} \qquad [36]$$

Applying these to the model we arrive at a system of equations for the probability of arriving at a node and it being present c_1^* and m_1^* which are very similar to the uncorrelated case.

$$\begin{aligned} c_1^* &= C_1(m_1^*), \\ m_1^* &= 1 - M_1(1 - c_1^*). \end{aligned} \qquad [37]$$

The probability of arriving at a present consumer is simply the probability of arriving at a consumer with a given indegree (accounting for the correlations) and all of those requirements being present. Likewise the probability of arriving at a metabolite and it being present is the probability we arrive at a node with a given degree and then any of the production links is present. We can also write the expressions for the probability of presence for a random node in terms of the new variables as

$$\begin{aligned} c^* &= C(m_1^*) \\ m^* &= 1 - M(1 - c_1^*) \end{aligned} \qquad [38]$$

which gives the full system of equations to solve. As before we can solve for c_1^* constructing a consistency equation from Eq. 37

$$c_1^* = C_1(1 - M(1 - c_1^*)) \qquad [39]$$

the solutions of which can be substituted into equations 37 and 38 to get c^* and m^*.

Random Correlated Graphs. To illustrate the patterns of diversity in correlated cross-feeding networks we consider the case of random graphs with correlations in node in- and outdegree. Whilst there is no single canonical bivarate Poisson distribution we use a simple formulation that lets us examine the effect of the correlation. Specifically we consider the joint in- and outdegree distribution (j, k) where

$$\begin{aligned} j &= j' + R \\ k &= k' + R \end{aligned} \qquad [40]$$

where j',k' and R are all Poisson random variables with parameters $z^{\text{in}} = z_j' - r$, $z^{\text{out}} = z_k' - r$ and r respectively. The dependence of the two degree distributions is determined by parameter for the shared term r

$$\text{Cov}(j, k) = r. \qquad [41]$$

The correlation is given by

$$\rho = \frac{r}{\sqrt{z^{\text{in}} z^{\text{out}}}}. \qquad [42]$$

The value of r is additionally constrained by $r < \min(z^{\text{in}}, z^{\text{out}})$ as the rate parameters must be positive. This sets an upper bound on the correlation based on the in- and outdegree.

$$\rho < \frac{\min(z^{\text{in}}, z^{\text{out}})}{\sqrt{z^{\text{in}} z^{\text{out}}}}. \qquad [43]$$

Deriving the generating function for the bivarate Poisson is simple using the definition of a multivariate generating function

$$G(x,y) = E[x^j y^k] \quad [44]$$

$$= E[x^{j'+R} y^{k'+R}] \quad [45]$$

$$= E[x^{j'}]E[y^{k'}]E[(xy)^R] \quad [46]$$

using the fact that j',k' and R are independent in the last step. As they are Poisson distributed each of the expectations follows the same form $F(x) = \exp(z(x-1))$ giving

$$G(x,y) = \exp\left[z^{\text{in}}(x-1) + z^{\text{out}}(y-1) + r(xy-1)\right] \quad [47]$$

we can confirm the marginal distributions still have a Poisson distribution using Eq. 30

$$G^{\text{in}}(x) = G(x,1) = \exp\left[(z^{\text{in}} + r)(x-1)\right] \quad [48]$$

Generating Correlated Poisson Variables In this section we discuss our method to generate random cross-feeding networks with degree correlations. As discussed in the methods, a major constraint on the degree distributions is that the number of start- and endpoints of links in the network must match for the structure to be feasible. The presence of correlations means that the links cannot be drawn independently and must be generated together.

To generate the networks we initialise the N consumers and M metabolites and first sample the consumer in- and out degrees from the bivariate Poisson distribution described in the previous section. We draw the degrees with desired marginal averages z_c^{in} and z_c^{out} and correlation ρ, with the additional constraint shown in Eq. 43.

By defining the in- and outdegree of the consumer nodes we have placed a double constraint on the resource degrees whose sum of out- and indegree must match accordingly. We can draw samples according to this constraint and a desired covariance (and thus correlation) by using the fact that a a Poisson sample conditioned on its sum has a multinomial distribution. First we write sums of the degree distributions as

$$\begin{aligned} S_c^{\text{in}} &= S_m^{\text{out}} = S_{k'} + S_r \\ S_c^{\text{out}} &= S_m^{\text{in}} = S_{j'} + S_r \end{aligned} \quad [49]$$

where S_c^{in} indicates the number of links entering consumers which must equal S_m^{out}, the number of links exiting metabolites. The last terms decompose the sums into the individual and shared components of the correlated Poisson in Eq. 40.

As we want to set the covariance externally we can obtain the distribution of S_r which as the sum of N Poisson variables with mean r (i.e the covariance; Eq. 41) will follow a Poisson distribution with mean Nr. Thus to sample the constrained distribution we first sample the value of S_r, use this to solve for $S_{k'}$ and $S_{j'}$. We then sample the distributions of k',j' and r from the correct multinomial distributions and sum them to get the individual joint degrees.

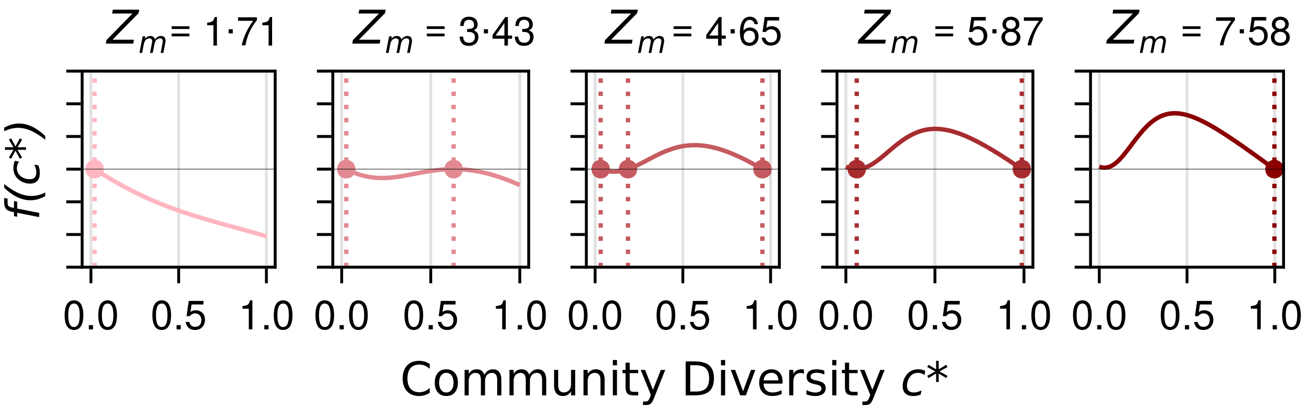

Fig. S1. Solutions to the self consistency equation Plot showing the self consistency equation across a range of average metabolite production degree whilst holding the population requirement constant at $z_c = 4.0$. Increasing z_m moving from left to right we see that below the critical point a single low-diversity solution exists. In the second panel at the critical value an additional solution appears. As z_m increases three solutions are present, two of which are stable. In panel 4 we again reach a critical point where a stable and unstable equilibrium collide. After this only the high-diversity state exists.

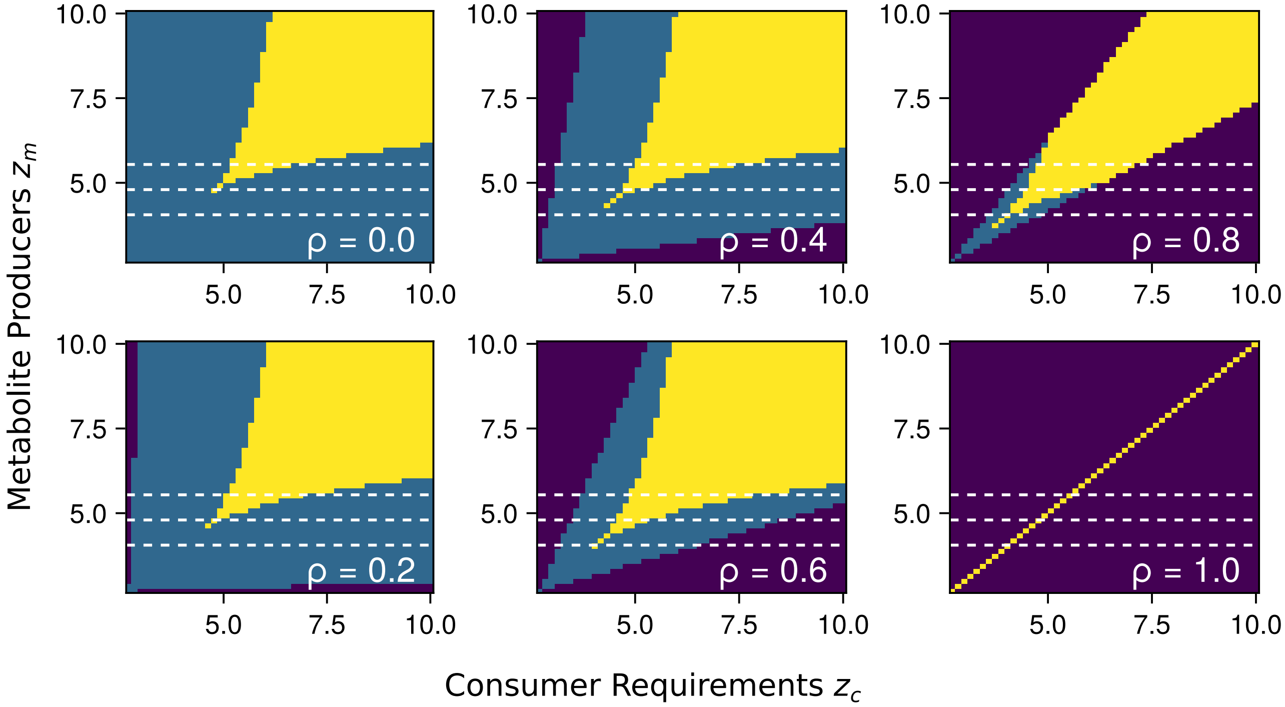

Fig. S2. Discontinuous transitions persist with degree correlations Phase plots showing how the cusp bifurcation persists in the presence of correlations in node degree. Panels show the bifurcation surface across different levels of correlation. Yellow areas indicated the "folded" region where multiple steady states coexist. Blue areas have a single solution and the black areas are unfeasible (i.e. the condition on ρ in Eq. 43 is not met). Overall increasing the correlations shifts the cusp bifurcation point to lower network degrees but also reduces the width of the bifurcation area. At high correlations the number of feasible average degree values is reduced till they must be equal $z_m = z_c$ when $\rho = 1$

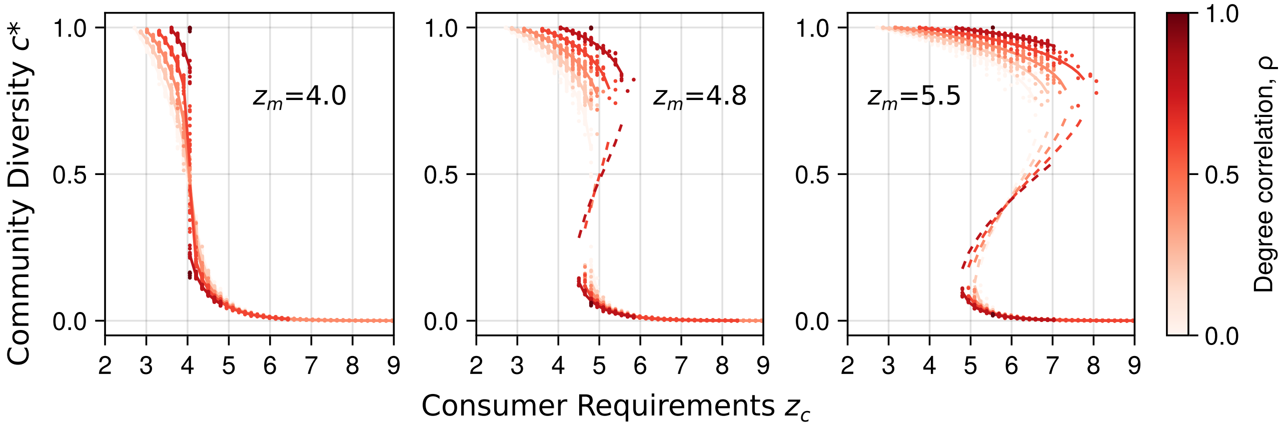

Fig. S3. The effect of degree correlations on community diversity Plots showing how community diversity changes with the correlation between in- and outdegree in the cross-feeding network. Lines show the diversity over differing numbers of requirements z_c on the x-axis and numbers of metabolite producers z_m across each panel, obtained from solutions to Eqs. 38 & 37. Colours indicate the strength of degree correlations ρ. Overall the analytical results match the generated networks very well. As the strength of correlations increases the region of path-dependency increases in size.

References

1. T Gross, L Barth, Network robustness revisited. *Front. Phys.* **10**, 823564 (2022).
2. ME Newman, SH Strogatz, DJ Watts, Random graphs with arbitrary degree distributions and their applications. *Phys. Rev. E* **64**, 026118 (2001).
